# The influence of patient factors on femoral rotation after total hip arthroplasty

**DOI:** 10.1186/s12891-018-2110-y

**Published:** 2018-06-09

**Authors:** Taro Tezuka, Yutaka Inaba, Naomi Kobayashi, Hyonmin Choe, Syota Higashihira, Tomoyuki Saito

**Affiliations:** Department of Orthopedic Surgery, Yokohama City University, 3-9 Fukuura, Kanazawa-ku, Yokohama, Japan

**Keywords:** Total hip arthroplasty, Femoral rotation, Femoral anteversion, Multiple regression analysis

## Abstract

**Background:**

A postoperative change in femoral rotation following total hip arthroplasty (THA) might be the cause of dislocation due to the change in combined anteversion. However, very few studies have evaluated the femoral rotation angle following THA, or the factors that influence femoral rotation. We aimed to evaluate changes in femoral rotation after THA, and to investigate preoperative patient factors that influence femoral rotation after THA.

**Methods:**

This study involved 211 hips treated with primary THA. We used computed tomography to measure the femoral rotation angle before and one week after THA. In addition, multiple regression analysis was performed to evaluate preoperative patient factors that could influence femoral rotation after THA.

**Results:**

The femoral rotation angle was 0.2 ± 14° externally before surgery and 4.4 ± 12° internally after surgery (*p* < 0.001). Multiple regression analysis revealed that sex (β = 0.19; *p =* 0.003), age (β = 0.15; *p =* 0.017), preoperative anatomical femoral anteversion (β = − 0.25; *p =* 0.002), and preoperative femoral rotation angle (β = 0.36; *p <* 0.001) were significantly associated with the postoperative femoral rotation angle. The final model of the regression formula was described by the following equation: [*postoperative femoral rotation angle* = 5.41 × *sex* (female: 0, male: 1) + 0.15 × *age* - 0.22 × *preoperative anatomical femoral anteversion* + 0.33 × *preoperative femoral rotation angle* - 10.1].

**Conclusion:**

The current study showed the mean internal change of 4.6° in the femoral rotation angle one week after THA. Sex, age, preoperative anatomical femoral anteversion and preoperative femoral rotation were associated with postoperative femoral rotation. The patients who were male, older, and who exhibited lesser preoperative anatomical femoral anteversion or greater preoperative femoral rotation angles, tended to demonstrate an externally rotated femur after THA. Conversely, patients who were female, younger, and who exhibited greater preoperative anatomical femoral anteversion or lesser preoperative femoral rotation angles, tended to demonstrate an internal rotation of the femur after THA.

## Background

Total hip arthroplasty (THA) is one of the most effective procedures for relieving arthritic pain caused by destructive hip diseases. However, despite improvements in prostheses, postoperative complications still occur. Among them, dislocation is a severe complication that sometimes leads to revision surgery [1, 2], and a meta-analysis has reported hip dislocation to be one of the common causes of THA failure [3]. Recently, postoperative changes in pelvic tilt have been reported, which cause angular changes of the acetabular components and dislocation [4–11]. A postoperative modification of the femoral rotation angle also might be the cause of dislocation due to the changes in combined anteversion; however, there were only three reports that investigated changes in femoral rotation after THA [12–14]. Uemura et al. reported that femurs rotated internally after THA [12, 14]. Akiyama et al. also found femoral internal rotation to occur after THA, and this rotation was associated with underlying disease, preoperative range of hip internal rotation, gender, surgical approach, leg lengthening, and a postoperative change in femoral anteversion [13]. Thus, the aims of the current study were to investigate the change of femoral rotation after THA and to investigate the preoperative patient factors that influenced femoral rotation after THA in our cohort.

## Methods

**Fig. 1:**
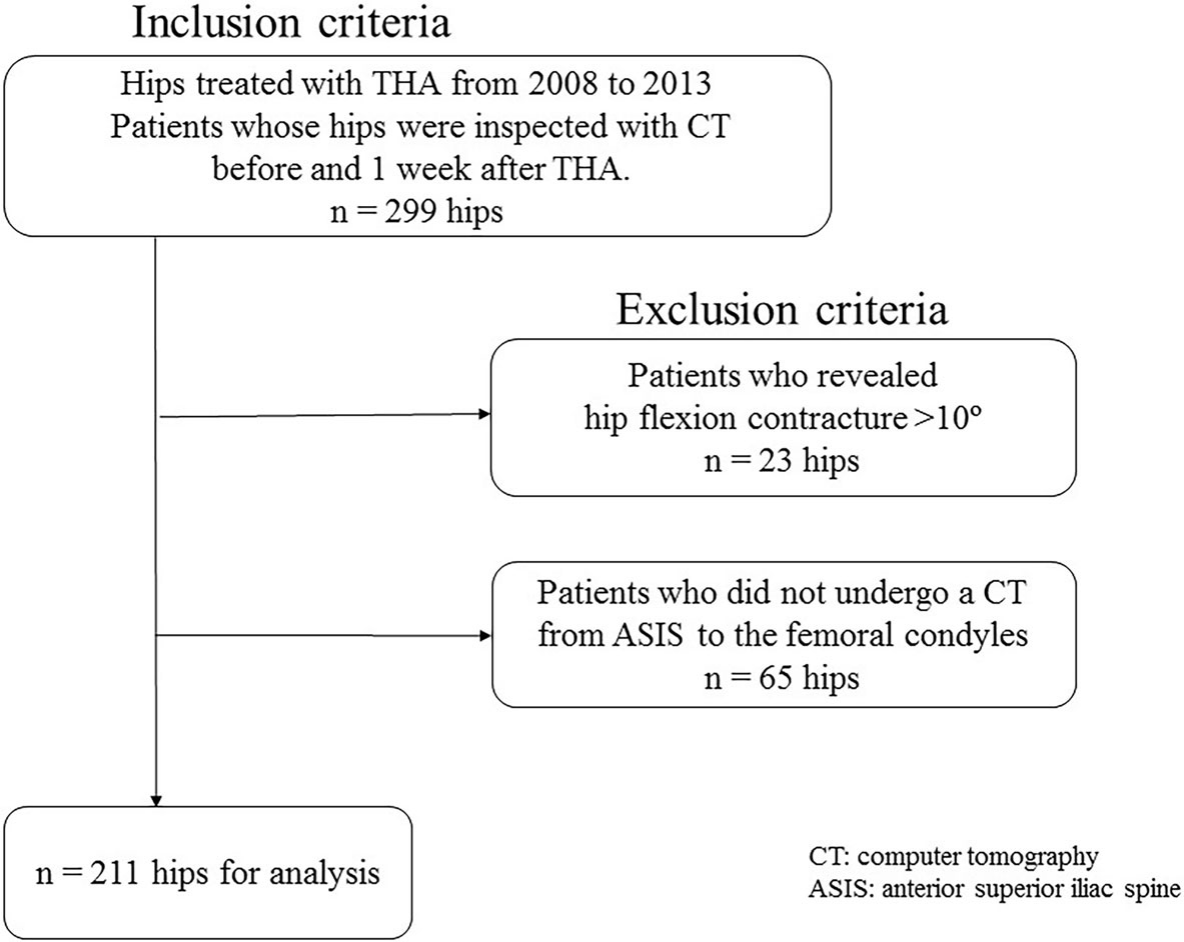
Inclusion and exclusion criteria of the study participants

The study was approved by the authors’ institutional review board of Yokohama City University (B170100008), and informed consent was obtained from all patients. Inclusion criteria for the current study were as follows: hips treated with THA between 2008 and 2013, and patients whose hips were examined using with computed tomography (CT) before and one week after THA. In total, 299 hips met the inclusion criteria. Exclusion criteria included a hip flexion contracture of more than 10 °, and absence of CT imaging from the anterior superior iliac spine (ASIS) to the femoral condyles; 23 and 65 patients were excluded, respectively. Therefore, 211 hips of 179 patients were enrolled in the current study (Fig. 1). These patients included 137 females and 42 males, and the mean age at surgery was 62 ± 12 years (range: 34–89 years). The mean body mass index (BMI) was 24 ± 4.0 kg/m^2^ (range: 14–35 kg/m^2^). The diagnoses were osteoarthritis in 164 hips (78%), osteonecrosis of the femoral head in 34 (16%), rheumatoid arthritis in 11 (5.1%) and subchondral insufficiency fracture of the femoral head in 2 hips (0.9%).

All surgeries were performed by two surgeons (YI and NK), and there was no difference in surgical approach, operative method and postoperative rehabilitation. One hundred and fifty-five hips were treated with a mini-direct lateral approach, and 56 hips were treated with an anterolateral approach.

The femoral prostheses used in the current study included 43 SL-PLUS™ stems (Smith and Nephew, Memphis, TN, USA); 53 SL-PLUS™ MIA stems (Smith and Nephew); 74 Accolade stems (Stryker Orthopedics, Mahwah, NJ, USA) with a 132 or 127 neck-shaft angle; 5 CentPillar stems (Stryker Orthopedics); 6 Exeter stems (Stryker Orthopedics); 1 Supersecure Fit stem (Stryker Orthopedics); 11 Taperloc stems (Biomet, Warsaw, IN, USA); 15 VerSys Fiber Metal MidCoat stems (Zimmer, Warsaw, IN, USA); and 3 VerSys Heritage stems (Zimmer). Surgeons decided to use each type of prosthesis in an attempt to optimize each patient’s kinematics based on preoperative radiographs and considering the proximal femoral bone quality or the presence of osteoporosis. More specifically, cemented stems were used for a patient with severe rheumatoid arthritis, severe osteoporosis, or a canal flare index ˂ 3. Acetabular prostheses used in the current study included 96 Reflection cups (Smith and Nephew); 75 Trident Acetabular cups (Stryker Orthopedics); 6 ADEPT cups (Stryker Orthopedics); 5 Crossfire all polyethylene cups (Stryker Orthopedics); 11 M2a Magnum cups (Biomet); 15 Trilogy cups (Zimmer); and 3 ZCA natural spacer cups (Zimmer).

All acetabular components except for the cemented cup were implanted with the use of the Stryker Navigation System II Cart with CT-based Hip Navigation software, version 1.1 (Stryker Orthopedics) or the Stealth Station Navigation System Treon Plus with Universal HIP software (Medtronic Inc., Minneapolis, MN, USA). Forty-three femoral components were implanted with the use of the Stryker Navigation System.

For preoperative planning, a combined anteversion of 37.3 ° was planned as derived from the equation, [*combined anteversion = cup anteversion* + *anatomical stem anteversion* × 0.7] as reported by Widmer et al. [15] in the functional pelvic plane (FPP) as described in our previous report [16].

### Clinical assessment

Each patient underwent careful visual assessment during gait and the evaluation for the presence of any discomfort or pain was performed during gait or physical examination of hip passive flexion, due to its association with hip impingement. This evaluation was performed at one week and at one year postoperatively. We also monitored the occurrence of hip dislocations during the study period.

### Radiographic assessment

**Fig. 2:**
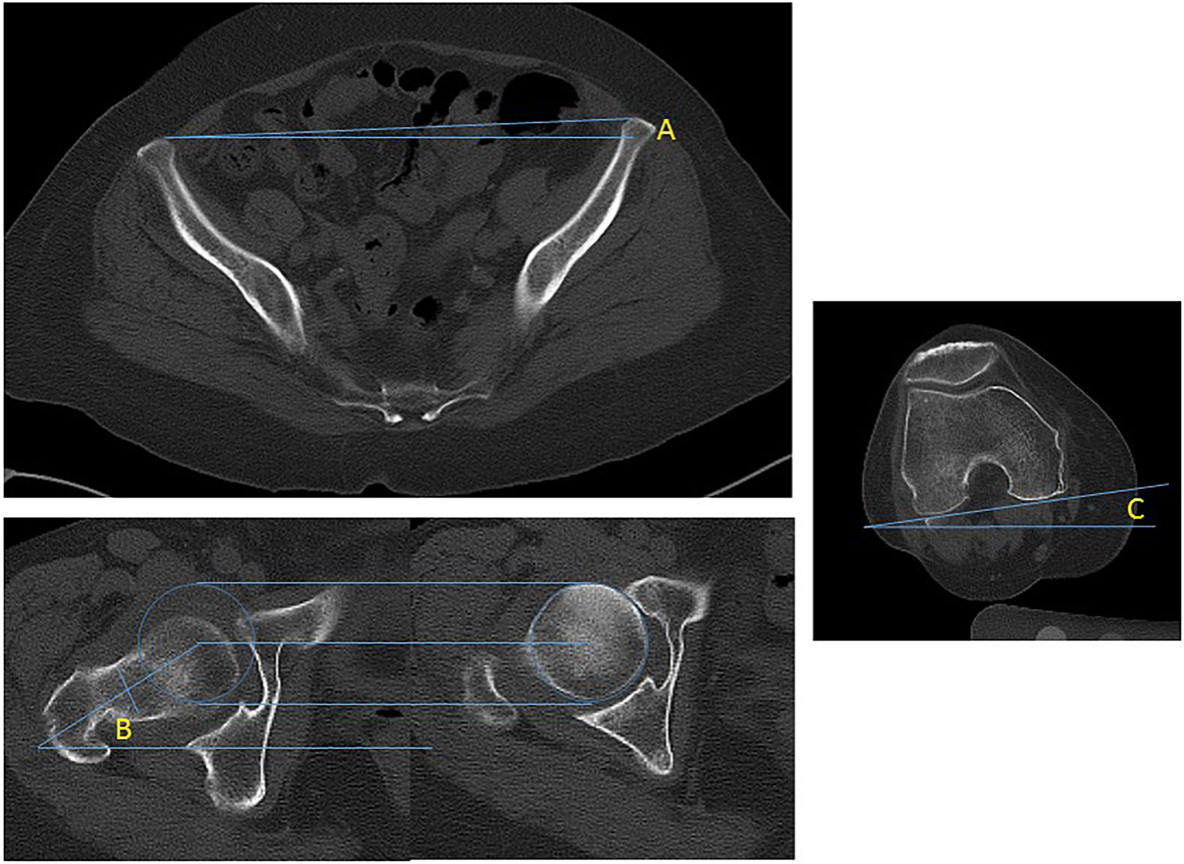
Measurements on pelvic and lower extremity axial computer tomography (CT). Angle **a** is defined as the angle between the line of the CT berths and the line connecting both anterior superior iliac spines. Angle **b** is defined as the angle between the line of the CT berths and the line connecting the femoral head center with the neck center. Angel **c** is defined as the angle between the line of the CT berths and the posterior condylar line

A CT from the pelvis to the distal femur was obtained before and one week postoperatively to make three-dimensional planning and to detect the presence of venous thromboembolism, and we evaluated the CT data for the current study. During the CT examination, the patient lay supine with the lower extremities in a relaxed position. In the CT evaluation, we defined the line between the anterior points of the bilateral ASISs as the ASIS line in the axial plane. The anterior pelvic plane included the ASIS line, and the ASIS line indicated the guidepost of pelvic rotation in the axial plane. Next, three angles were measured. Angle A was defined as the angle between the line of the CT berths and the ASIS line and was considered positive if the pelvis rotated towards the affected side, and negative if the pelvis rotated towards the healthy side. Angle B was defined as the angle between the line of the CT berths and the line connecting the femoral head center with the femoral neck center. Angle C was defined as the angle between the line of the CT berths and the posterior condylar line (PCL) (Fig. 2). The femoral rotation angle was defined as the angle between the PCL and the ASIS line and was calculated as Angle C - Angle A. The femoral rotation angle was positive if the femur rotated externally, and negative if the femur rotated internally. The anatomical femoral anteversion was defined as the angle between the PCL and the line connecting the femoral head and neck centers and was calculated as Angle B - Angle C. The functional femoral anteversion was the angle between the ASIS line and the line connecting the femoral head and neck centers and was calculated as Angle B - Angle A. From the postoperative CT images, we calculated the femoral rotation angle, the anatomical stem anteversion and the functional stem anteversion in the same manner. Among 211 hips, we also measured the femoral rotation angle at one year after surgery using CT images of 25 hips that were taken for other studies [17, 18]. We compared the values with those of one week after THA to evaluate the time-course change in rotation after the surgery. At one year after THA, anteroposterior and lateral radiographs of the hip were taken to detect any abnormal findings that indicated the femoral component loosening, such as progressive axial subsidence more than 3 mm or varus or valgus migration or the presence of a complete radiolucent line surrounding femoral components.

Radiographic measurements were taken two times after an interval of at least two weeks by a single author, and intra-observer reliabilities of the radiographic measurements were assessed using intra-class correlation coefficients. The average values of the radiographic measurements were calculated and analyzed. For the inter-observer reliability, TT and SH assessed each of the measurements using inter-class correlation coefficients, which can range from − 1 to 1, with 1 indicating perfect agreement. Table 1 shows the intra- and inter-class correlation coefficients for each measurement, which were considered excellent.

**Table 1:** The intra- and interclass correlation coefficients for each radiographic measurement

	Angle A		Angle B		Angle C	
	Preop	Postop	Preop	Postop	Preop	Postop
Intraclass CC	0.92	0.93	0.90	0.97	0.95	0.98
Interclass CC	0.91	0.92	0.90	0.97	0.95	0.97

*CC* correlation coefficient, *Preop* preoperative, *Postop* postoperative

### Statistical analysis

The paired *t*-test was used to evaluate the differences in each radiographic measurement (pre- and post- femoral rotation angle, anatomical femoral or stem anteversion, and functional femoral or stem anteversion). To evaluate differences in radiographic variables between groups treated by different surgeons, the unpaired *t*-test was performed. Analysis of variance (ANOVA) was performed to test the differences among groups with different types of stems.

To analyze the factors that influence the femoral rotation angle after THA, a regression analysis was performed. At first, a simple regression analysis was performed with the following factors as explanatory variables: sex, age, BMI, preoperative anatomical femoral anteversion and preoperative femoral rotation angle. Furthermore, variables with *p*-values < 0.05 were populated for regression analysis as a multivariate assessment and entered into the final equation. The adjusted coefficient of multiple determination (adjusted R^2^) was used to indicate how much variability in the femoral rotation angle after THA was accounted for by the independent variables. Standardized regression coefficients (β) and associated *p*-values were determined to assess statistical significance (*p <* 0.05). All statistical analyses were performed using SPSS, version 21.0 (IBM Corp., Armonk, NY, USA).

## Results

No patients had complaints one week after surgery except pain originating from the incision scar and there were no symptoms of impingement or gait abnormalities that could be clearly associated with excessive femoral rotation. All patients could walk independently with or without a cane and were discharged from the hospital at 10 days after THA. At one year after surgery, there were no complaints of gait abnormalities. No dislocations were observed throughout the study period.

The mean preoperative femoral rotation was 0.2 ± 14° and the mean postoperative femoral rotation was − 4.4 ± 12° (*p* < 0.001) (Table 2). Among 211 hips, 88 hips (41.7%) had internally rotated femurs before and after THA, and 43 hips (20.4%) had femurs rotated externally before THA and internally after THA. Twenty hips (9.5%) showed femoral internal rotation before THA and external rotation after THA. Fifty-three hips (25.1%) demonstrated femurs rotated externally before and after THA. (Table 3). The mean preoperative femoral rotation angle of 110 hips showing internal rotation before THA was − 11 ± 7.9 ° and the mean femoral rotation angle of 97 hips that revealed an external rotation before THA was 12 ± 8.1 °. The mean femoral rotation angle of 134 hips that revealed internal rotation after THA was − 11.7 ± 7.5 ° and among the 134 hips, 17 hips showed the femoral rotation angle less than − 20 °. The mean femoral rotation angle of 74 hips showing external rotation after THA was 8.5 ± 7.3 ° and among the 74 hips, there were 6 hips revealed the femoral rotation angel more than 20 °. The preoperative anatomical femoral anteversion was 22 ± 14°, and the postoperative anatomical stem anteversion was 31 ± 14° (*p* < 0.001) (Table 2), which indicated the surgeons increased stem anteversion by 9° during THA. The preoperative functional femoral anteversion was 22 ± 12°, and the functional stem anteversion was 27 ± 13° (*p* < 0.001) (Table 2). These results indicated that functional stem anteversion increased by 5° after THA, while the surgeons increased anatomical stem anteversion by 9°. The difference between pre- and postoperative anatomical anteversion was larger than the difference between pre- and postoperative functional anteversion (*p* < 0.001); this was caused by changes in femoral rotation. There was no significant difference in radiographic variables between groups treated by different surgeons, and ANOVA testing showed that there was no significant difference in radiographic variables among groups with different types of femoral implants.

**Table 2:** The results of the radiographic measurements

	Preop (range)	Postop (range)	*p*
Femoral rotation angle (°)	0.2 ± 14 (− 42–37)	−4.4 ± 12 (− 32–32)	< 0.001
Anatomical femoral (stem) anteversion (°)	22 ± 14 (− 14–63)	31 ± 14 (− 11–59)	< 0.001
Functional femoral (stem) anteversion (°)	22 ± 12 (− 11–66)	27 ± 13 (− 2–70)	< 0.001

*Preop* preoperative, *Postop* postoperative

**Table 3:** The number of hips in each position pre- and post-surgery

		Preop			
		Internal rotation	Neutral	External rotation	Total
Postop	Internal rotation	88 (41.7%)	3 (1.4%)	43 (20.4%)	134
	Neutral	2 (0.9%)	0 (0%)	1 (0.5%)	3
	External rotation	20 (9.5%)	1 (0.5%)	53 (25.1%)	74
	Total	110	4	97	211

Preop: preoperative, Postop: postoperative

**Fig. 3:**
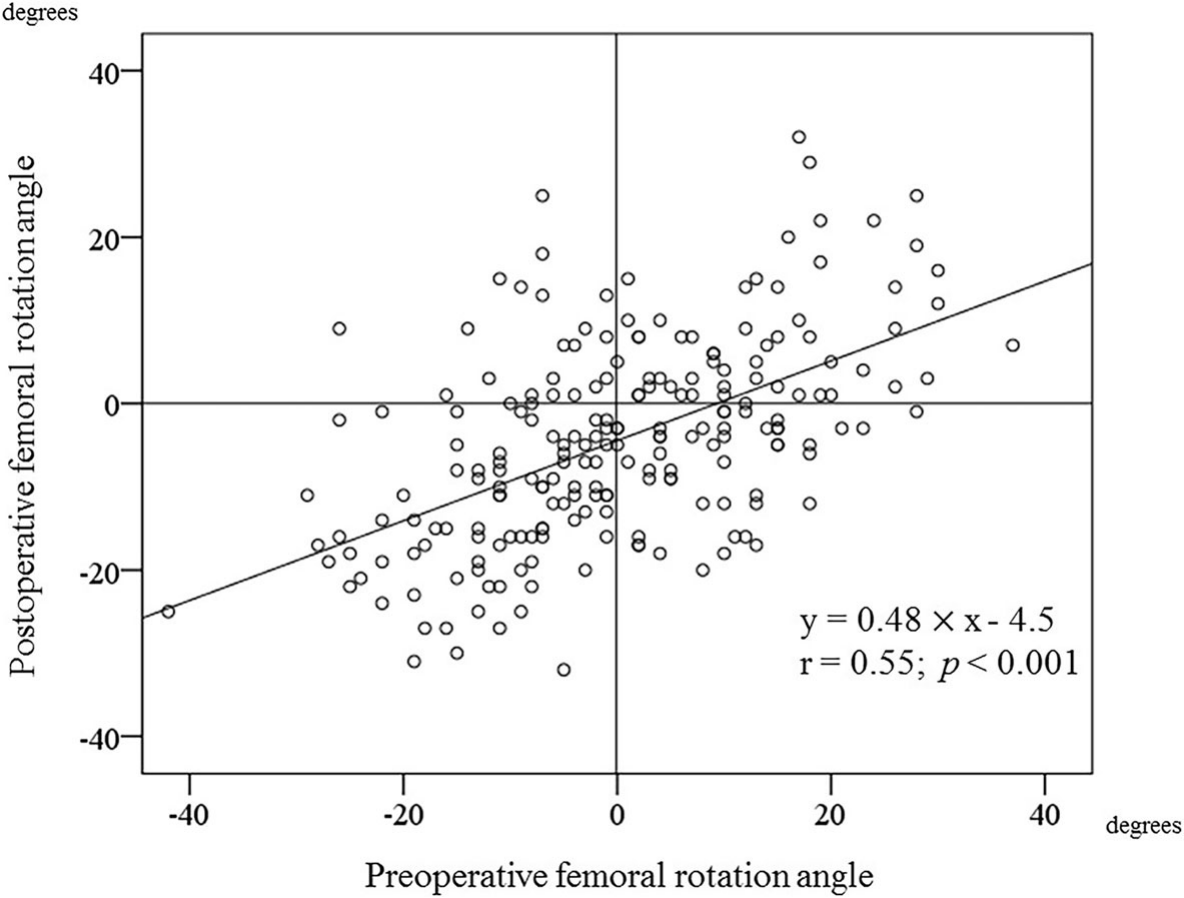
The scatter chart of pre- and postoperative femoral rotation angles. The X axis is the preoperative femoral rotation angle, and the Y axis is the postoperative femoral rotation angle. There is a positive correlation between the preoperative femoral rotation angle and the postoperative femoral rotation angle (r = 0.55; *p <* 0.001)

Table 4 shows the results of the univariate and multiple regression analyses. The simple regression analysis showed that sex, age, preoperative anatomical femoral anteversion and preoperative femoral rotation angle had a significant influence on the postoperative femoral rotation angle. Figure 3 shows a scatter plot of pre- and postoperative femoral rotation angles. There was a positive correlation between preoperative and postoperative femoral rotation angles (*r* = 0.55; *p <* 0.001). The final model of the multiple regression analyses revealed that sex (β = 0.19; *p* = 0.003), age (β = 0.15; *p* = 0.017), preoperative anatomical femoral anteversion (β = − 0.25; *p* = 0.002), and preoperative femoral rotation angle (β = 0.36; *p <* 0.001) were significantly associated with the postoperative femoral rotation angle (Table 4). The results of multiple regression analyses indicated that patients who were male, older, and exhibited lesser preoperative anatomical femoral anteversion or greater preoperative femoral rotation angles tended to show an externally rotated femur after THA. Conversely, the patients who were female, younger, and had greater preoperative anatomical femoral anteversion or lesser preoperative femoral rotation angles, tended to demonstrate an internal rotation of the femur after THA. The final model of the regression formula was obtained as the following equation: [*the postoperative femoral rotation angle* = 5.41 × *sex* (female: 0, male: 1) + 0.15 × *age* - 0.22 × *preoperative anatomical femoral anteversion* + 0.33 × *preoperative femoral rotation angle* - 10.1]. The final model accounted for 40% of the variation in postoperative femoral rotation angle (adjusted R^2^ = 0.40).

**Table 4:** The results of regression analysis

Univariate regression analysis				
		Regression coefficient	β	*p*
Sex (female:0, male:1)		5.35	0.19	0.006
Age		0.24	0.23	0.001
Body Mass Index		0.34	0.11	0.122
Surgical approach		0.73	0.03	0.697
Preoperative anatomical femoral anteversion		−0.46	−0.55	< 0.001
Preoperative femoral rotation angle		0.48	0.55	< 0.001
Multivariate regression analysis				
	Adjusted R^2^	Regression coefficient	β	*p*
Sex (female:0, male:1)	0.40	5.41	0.19	0.003
Age		0.15	0.15	0.017
Preoperative anatomical femoral anteversion		−0.22	−0.25	0.002
Preoperative femoral rotation angle		0.33	0.36	< 0.001

The average femoral rotation angle of 25 hips whose CT images were available at one week and one year after THA was − 5.5 ± 12 ° and − 11 ± 12 ° respectively (*p* < 0.001). There was a strong positive correlation between measurements of femoral rotation angles taken one week and one year after surgery (*r* = 0.80; *p* < 0.001). There were no findings that could indicate the femoral component loosening.

## Discussion

It is well-known that the orientation of acetabular and femoral components is one of the key factors affecting the THA success [19, 20]. From a historical aspect, the so-called “safe zone” of the acetabular cup angle is one of the crucial notions in reducing dislocation risk after THA [21]. Subsequently, the concept of “combined anteversion,” which is the sum of the acetabular and femoral anteversion, has been proposed for the decreasing dislocations and increasing impingement-free range of motion [15, 22, 23].

Several studies have reported on the relation between pelvic tilt and acetabular component angles [11, 24–28]. In general, the pelvis tilts posteriorly from supine to standing and with time after THA, especially in older patients whose lumbo-lordotic angle is smaller [4]. Considering this change in pelvic tilt and angle of acetabular components, the usability of the FPP has been reported [16, 29, 30]. However, there are few studies regarding the femoral rotation angle after THA [12, 14], and a limited number of reports have evaluated which patient factors affect the femoral rotation angle after THA [13].

In the current study, there was no patient who had complaints, abnormal gait or dislocation clearly due to the femoral rotation, however, there were 17 hips showed the femoral rotation angle of femur less than − 20 ° and 6 hips showed external rotation more than 20 °. Until now, there were no reports about established methods to prevent excessive femoral rotation after THA. Akiyama et al. reported that the internal rotation of the hip after THA increased with posterolateral approach which damages short rotators [13]. To use posterior approach might be one of effective procedures to prevent the excessive external rotation for hips that were considered to have external rotation after THA.

Akiyama et al. also reported that the femoral internal rotation showed an average increase of 11° after THA, while the current study showed the mean change in the femoral internal rotation angle after THA was 4.6°. They performed 82% of their THAs using the posterior approach [13], and all THAs in the current study were performed with a mini-direct lateral approach or anterolateral approach. This might be the cause of difference in the femoral rotation angle compared to the report of Akiyama et al. Furthermore, this fact also might be the reason that the surgical approach did not influence femoral rotation after THA in the current study.

There was a strong positive correlation between the preoperative and postoperative femoral rotation angles, and regression analysis revealed that the preoperative femoral rotation angle was a significant factor in predicting postoperative femoral rotation. In addition to preoperative femoral rotation, sex, age and preoperative anatomical femoral anteversion displayed a significant effect on postoperative femoral rotation angle; therefore, a preoperative plan might be made with consideration of patient preoperative characteristics. In particular, patients who were male, older and had an externally rotated femur or smaller preoperative anatomical femoral anteversion tended to show an externally rotated femur after THA, which might cause the impingement that lead to anterior dislocation. In contrast, patients who were female, younger and had an internally rotated femur or larger preoperative anatomical femoral anteversion tended to show an internally rotated femur that might result in posterior dislocation. In such cases, the results of the current study suggest that femoral prostheses should be implanted with less or more degree of anteversion, derived from the following equation; [5.41 × *sex* (female: 0, male: 1) + 0.15 × *age* - 0.22 × *preoperative anatomical femoral anteversion* + 0.33 × *preoperative femoral rotation angle* - 10.1].

Among the 211 hips, 25 hips underwent CT at one year after THA. The average femoral rotation angle was significantly different at one week and at one year after THA (− 5.5 ± 12° and − 11 ± 12°, respectively, *p* < 0.001) with a significant and strong correlation between them. The result of one year after THA was quite same as the result of Akiyama et al. [13]. Uemura et al. also reported that substantial variability was observed in the femoral rotation angle, especially during the postoperative period [14]. We could not identify what factors would affect the change in the femoral rotation angle between one week and one year after THA in the current study, but one of the reasons might be recovery of muscles around the hip joint after THA. The result of the current study indicated that the femoral rotation left the possibility of changing internally by a mean of 5 ° until one year after THA.

To the best of authors’ knowledge, this is the first study to propose an equation to predict postoperative femoral rotation after THA using preoperative patient factors. However, there are several limitations to the current study. First, radiographic measurements were performed on the two-dimensional axial plane of a CT image, rather than via a three-dimensional reconstructed model. However, the measurement technique was consistent, and patients who showed any flexion contracture of more than 10° were excluded. Inter- and intraclass correlation coefficients were sufficiently high. Thus, we believe the data in the current study was reliable. Second, the evaluation was focused only on the supine position. Postural changes including standing or sitting were not considered, and further research should be address these factors.

From the results of the multiple regression analysis, we obtained an equation with which we could partially predict the postoperative femoral rotation using patient preoperative factors, and the equation might be helpful in determining femoral implant anteversion. The adjusted R^2^ obtained from the multiple regression analysis was 0.40, which indicates there were other factors that significantly affected the femoral rotation. Akiyama et al. reported that the femoral rotation after THA was not only associated with certain pre- and perioperative factors, that is, underlying disease, pre-operative range of hip internal rotation, gender, surgical approach, but also with postoperative factors including leg lengthening and change of femoral anteversion [13]. Because the aim of the current study was to investigate the preoperative patient factors that influenced the femoral rotation after THA, we chose not to consider postoperative patient factors; however, these factors must have an influence on femoral rotation. Further investigation into the relationships between femoral rotation and other factors may be required.

## Conclusions

The current study showed the mean internal change of 4.6° in the femoral rotation angle one week after THA. There was no significant difference in femoral rotation between groups treated with a mini-direct lateral approach and treated with an anterolateral approach. Sex, age, preoperative anatomical femoral anteversion, and preoperative femoral rotation angle were significant predictors for the postoperative femoral rotation angle in the supine position.

## Abbreviations

adjusted R^2^: Adjusted coefficient of multiple determination
ASIS: Anterior superior iliac spine
BMI: Body mass index
CT: Computed tomography
PCL: Posterior condylar line
THA: Total hip arthroplasty
β: Standardized regression coefficients
